# Androgen Receptor: A Complex Therapeutic Target for Breast Cancer

**DOI:** 10.3390/cancers8120108

**Published:** 2016-12-02

**Authors:** Ramesh Narayanan, James T. Dalton

**Affiliations:** 1 Department of Medicine, University of Tennessee Health Science Center, Memphis, TN 38103, USA; rnaraya4@uthsc.edu; 2 College of Pharmacy, University of Michigan, Ann Arbor, MI 48109, USA

**Keywords:** androgen receptor, breast cancer, selective androgen receptor modulator (SARM), estrogen receptor, triple-negative breast cancer (TNBC)

## Abstract

Molecular and histopathological profiling have classified breast cancer into multiple sub-types empowering precision treatment. Although estrogen receptor (ER) and human epidermal growth factor receptor (HER2) are the mainstay therapeutic targets in breast cancer, the androgen receptor (AR) is evolving as a molecular target for cancers that have developed resistance to conventional treatments. The high expression of AR in breast cancer and recent discovery and development of new nonsteroidal drugs targeting the AR provide a strong rationale for exploring it again as a therapeutic target in this disease. Ironically, both nonsteroidal agonists and antagonists for the AR are undergoing clinical trials, making AR a complicated target to understand in breast cancer. This review provides a detailed account of AR’s therapeutic role in breast cancer.

## 1. Introduction

Over 240,000 women will develop breast cancer and ~40,000 will die from the disease in the United States in 2016 [1]. Globally, about 1.7 million women were diagnosed with breast cancer in 2012, emphasizing the urgent need for effective and safe therapeutic approaches [2]. Although the majority of breast cancers are slow growing or indolent [3], a subset acquires an aggressive phenotype due to a variety of reasons. Molecular, genotypic, and phenotypic studies clearly provide evidence for the heterogeneity of breast cancer with multiple subtypes and classifications [4,5].

## 2. Breast Cancer Classification

For therapeutic purposes, breast cancer has been historically classified based on the expression or lack of expression of estrogen receptor (ER), progesterone receptor (PR), and human epidermal growth factor receptor (HER2) [6]. Breast cancers expressing these three targets are classified as triple-positive, while those that lack their expression are classified as triple-negative (TNBC).

In 2000, Perou et al. completed a genome-wide molecular analysis of patient specimens to classify breast cancer based on cell-type and molecular signature [4]. Breast cancer specimens that expressed keratin 8/18, markers of luminal epithelial cells, were classified as luminal breast cancers, while those that expressed keratin 5/6, markers of basal epithelial cells, were classified as basal breast cancer. Further, using gene expression signatures, breast cancers were classified into luminal A, luminal B, HER2-enriched, and basal-like (BLBC).

The luminal A subtype is characterized by the expression of ER, lack of HER2, and a lower expression of the proliferative marker, Ki67 (ER+/HER2-/Ki67 low). The luminal A subtype is an indolent disease that is typically treated with hormonal therapies that either antagonize or degrade ER or inhibit aromatase, an enzyme critically involved in biosynthesis of estradiol.

The luminal B subtype is characterized by the expression of ER, lack of HER2, and high Ki67 (ER+/HER2-/Ki67 high). Although luminal B is predominantly HER2-negative, a subset of it expresses HER2 while still retaining other characteristics of HER2-negative luminal B. Markers of proliferation such as cyclin B1 (CCNB1), Ki67 (MKI67), and Myb proto-oncogene like 2 (MYBL2) [7,8] and proliferative growth factor signaling [9,10] are highly expressed in the luminal B subtype. The luminal B subtype is associated with high recurrence, poor disease-free survival [7] with much lower five- and ten- year survival rates than the luminal A subtype [7,11,12], and failure to respond consistently to any existing treatments [13].

The HER2 subtype is comprised of tumors that are ER-negative and HER2-positive [4]. This subtype is treated with HER2 inhibitors such as traztuzumab. The HER2 subtype frequently metastasizes to brain [14], escaping further inhibition by HER2-targeting antibodies that seldom cross the blood-brain barrier due to their large size.

The BLBC subtype is the most aggressive subtype of breast cancer and is associated with high mortality in women. While 75%–80% of the basal subtype is TNBC, the remaining 20%–25% express ER and/or HER2 [15]. It is still regarded as TNBC for therapeutic purposes and treated with a cocktail of chemotherapeutic agents that provide a pCR of about 40%–45% [16]. The cancer genome atlas (TCGA) studies indicate that the basal subtype has several features, including a high percentage of p53 mutations that confer an ovarian cancer phenotype rather than breast cancer [17].

## 3. TNBC Sub-Classification

Genome-wide studies to understand the underlying mechanisms for the aggressive phenotype of TNBC and to identify new therapeutic targets led to the classification of TNBC into six subtypes [5], including: Basal-like (BL1 and BL2) subtypes that are enriched in genes representing cell cycle, cell division, and DNA damage response. These two subtypes also express high levels of Ki67 at about 70% compared to 42% for other subtypes. Immunomodulatory (IM) subtype that is enriched in genes representing immune cell signaling. Mesenchymal (M) and mesenchymal stem cell like (MSL) subtypes that are enriched in pathways involved in cell motility, kinases, and differentiation. Luminal Androgen Receptor (LAR) subtype with high expression of Androgen Receptor (AR) mRNA and enrichment of hormonal signaling.

This subtyping provides an opportunity to develop focused therapeutics and conduct clinical trials in which the subjects belong to a particular subtype.

## 4. Androgen Receptor

The AR is a member of the nuclear hormone receptor family of ligand-activated transcription factors that is activated by androgen (i.e., testosterone or its locally synthesized and more potent metabolite, dihydrotestosterone (DHT). The AR gene is located on the X chromosome at q11 and contains eight exons encoding for an N terminus domain (NTD), a DNA binding domain (DBD), a hinge region, and a ligand binding domain (LBD). The NTD contains the activation function 1 domain (AF-1) that retains most of the AR activity [18]. The DBD contains two zinc finger motifs that recognize consensus androgen response elements (AREs) and anchoring of the AR to recognized sequences [19]. The hinge region is responsible for nucleo-cytoplasmic shuttling of the AR and the LBD that contains the ligand binding pocket is important for ligand recognition. The LBD of the AR contains 11 helices (unlike other receptors that contain 12 helices as the AR lacks helix 2) and the AF-2 domain [20].

The unliganded AR is maintained in an inactive complex by heat shock proteins, HSP-70 and HSP-90. Upon ligand binding, the HSPs dissociate from the AR enabling it to translocate into the nucleus and bind to DNA elements that are located both proximal and distal to the transcription start site [21]. Once bound to DNA, the AR recruits coactivators and general transcription factors to alter the transcription and translation of the target genes. While agonists recruit coactivators to augment transcription and translation of target genes, antagonists either recruit corepressors, prevent coactivators from associating with the AR, or retain the AR in the cytoplasm resulting in inactive AR.

## 5. Prognostic Value of the AR in Breast Cancer

Perhaps surprisingly, the AR is the most widely expressed nuclear hormone receptor in breast cancer with about 85%–95% of the ER-positive and 15%–70% of the ER-negative breast cancers expressing AR. In a study conducted with 2171 patient specimens, AR was found to be expressed in 77% of invasive breast carcinomas [22]. About 91% of the luminal A subtype tumors were positive for the AR, while 68% of the luminal B and 59% of the HER2 subtypes were positive for the AR. In addition, 32% of BLBCs expressed the AR in this cohort of 2171 patient specimens [22]. Interestingly, the study found an inverse correlation between the AR expression and tumor size, lymph node status, and histological grade. A higher proportion of the AR-positive tumors had smaller size compared to AR-negative tumors (24.6% vs. 15.8% for tumors less than 1 cm). Similarly, the majority of the AR-negative tumors were histological grade 3 tumors, while AR-positive tumors typically were histological grades 1 and 2 [22].

A review of a database containing data from 19 studies with a total of 7693 women demonstrated that the AR is expressed in 61% of the patients [23]. While 75% of the ER-positive tumors expressed AR, only 32% of the ER-negative breast cancers expressed the AR [23]. Tumors that expressed the AR were associated with improved overall survival (OS) and disease-free survival (DFS) compared to AR-negative tumors [23]. Considering the significance in this finding, the authors recommended that the AR be considered as one of three prognostic markers to classify breast cancers as triple-positive (ER, HER2, and AR-expressing) or triple-negative (ER, HER2, and AR-negative). Since *PR* is an ER-target gene, *PR* is most likely to align with ER expression pattern and hence was logical to exclude from the list of prognostic markers. These results were reproduced in other studies conducted in different patient cohorts around the world [24,25,26,27,28,29], including one clearly showing that expression of the AR was associated with reduced recurrence of the disease and reduced incidence of death in TNBC [28].

Noh et al. included 334 ER-negative HER2-positive or -negative breast cancers in a study to evaluate the expression of AR and clinical outcome [30]. Most of the AR-negative breast cancer patients were younger and had higher Ki67 compared to AR-positive breast cancer patients. While 27% of the TNBC patients were AR-positive, 53% of the ER-negative HER2-positive patients were AR-positive. Metabolic markers such as carbonic anhydrase (CAIX), which are associated with shorter DFS and OS, were significantly lower in AR-positive TNBC and ER-negative tumors [30].

One of the breast cancer subtypes where AR’s prognostic value was debated is the molecular apocrine type [31]. Molecular apocrine breast cancers, which constitute about 5%–10% of the breast cancers, are ER- and PR- negative [31,32]. The lack of these hormone receptors makes them unresponsive to associated hormonal therapies. One of the unique features of the molecular apocrine breast cancers is that they express AR, potentially making AR a valuable prognostic and therapeutic target [5]. Since AR and androgens increase the proliferation of a molecular apocrine breast cancer cell line, MDA-MB-453, it is widely perceived, albeit falsely, that AR is an unfavorable therapeutic target and prognostic marker in molecular apocrine subtype [33,34]. However, a study compared 20 molecular apocrine cancers with 26 non-apocrine cancers for AR expression and other clinical features [35]. All apocrine carcinomas were AR-positive, while all non-apocrine tumors were AR-negative. While apocrine tumors had grades between G1 and G3 and low T stage (TNM classification where T corresponds to tumor size), all non-apocrine tumors were G3 and high T stage. In addition, 80% of the apocrine tumor patients showed no disease-related mortality. These results present additional evidence to support the idea that the AR is a good prognostic marker with potentially favorable function in breast cancer.

In addition to measuring AR expression, some studies measured the expression of androgen-synthesizing enzymes such as 17βHSD5 (also known as AKR1C3) and 5α-reductase. 17βHSD5 converts the weaker androgen, androstenedione, to a more potent testosterone, while 5α-reductase further amplifies the activity by converting testosterone to the more highly potent DHT [36]. McNamara et al. evaluated 203 TNBC specimens from Thailand and Japan in a study to measure the expression of the AR and androgen-synthesizing enzymes [37]. While 25% of the patients were AR-positive, 72% were 5α-reductase-positive and 70% were 17βHSD5-positive. AR expression inversely correlated with Ki67 staining. Co-expression of the AR and androgen-synthesizing enzymes negatively correlated with Ki67 staining. Although no significant improvement in OS and DFS was observed in the AR- and 5α-reductase- positive cohort, the AR-negative 5α-reductase-positive cohort had worse survival in an 80 month follow-up.

A recent study evaluated the expression of AR and other genes in 1141 patient specimens [38]. Nuclear AR expression, which is an indirect measure of activated AR, was associated with favorable prognosis such as smaller tumor size, lower grade, and overall survival, suggesting that AR activation is favorable in breast cancer [38]. These observations were more pronounced in the luminal breast cancer subtypes [38].

An overwhelming number of publications demonstrate that the AR is a favorable prognostic marker (i.e., that the AR is a protective protein), regardless of the tumor subtype, and suggest that in most, if not all, cases AR expression is inversely proportional to tumor size, aggressiveness, pathological grade, and directly proportional to DFS, progression-free survival (PFS), and OS. However, a few reports have identified a subset of cancers where AR expression is directly proportional to Ki67 staining and correlates with poorer OS and DFS [39,40]. For example, a study conducted in a Chinese cohort of 450 breast cancer patients [40] showed that AR expression correlated with an increase in DFS in luminal breast cancer patients but a decrease in DFS in patients with TNBC. These results further illustrate the complex role of the AR in breast cancer. This information is summarized in Table 1.

## 6. AR as Predictor of Therapeutic Response

While the above studies strongly suggest that AR expression predicts favorable prognosis, AR expression also provides information on the treatment response. In a study evaluating 913 patients, AR expression was associated with a favorable outcome to treatment [41]. Patients with tumors that expressed ER, but not AR, failed aromatase inhibitor (AI) therapy earlier. Since aromatase converts testosterone to estradiol, inhibiting the enzyme will potentially increase intracellular testosterone, an AR agonist. This observation suggests that activation of the AR is an important factor for sustained therapeutic outcome with AI. In addition to the above study, an interesting observation [42] indicated that patients with AR-positive tumors benefited from tamoxifen treatment, whereas patients with AR-negative tumors had worse outcome.

Loibl et al. evaluated 673 core primary breast cancer biopsies from patients who have received neoadjuvant chemotherapy [43]. AR was detected in 53% of the entire cohort with 67% in luminal A and 21% in TNBC. Similar to several other studies, AR expression correlated with better DFS and OS in both luminal breast cancer and TNBC. However, the pathological complete response (pCR) in the AR-positive group was only 13%, which is similar to rates observed for the luminal A subtype, compared to 25% in the AR-negative cohort, which is similar to rates observed in the luminal B or TNBC subtype. This data indicates that the AR-negative cohort had a better chance of attaining pCR and provides evidence that, regardless of the breast cancer subtype and ER/PR/HER2 expression, AR-expressing tumors appear to retain the characteristics of the luminal A subtype when responding to chemotherapeutic agents. This hypothesis was corroborated by other studies. Lehmann et al. in their TNBC sub-classification study found that the LAR subtype of the TNBC expressed a luminal gene expression pattern including luminal markers such as FOXA1, KRT18, and XBP1 [5]. Indolent AR-positive luminal A subtype has a pCR of only 10% in response to chemotherapeutic agents, while the BLBC or TNBC tumors have approximately 50% pCR [16,44]. In addition, out of the six molecular subtypes in TNBC, basal-like is the only subtype that provided a significant association between pCR and survival after chemotherapy [45].

## 7. Role of Intracrine Androgen Synthesis in Breast Cancer

Intracrine hormone synthesis in breast and prostate cancers has been recognized in the recent years as a vital but previously unrecognized driver of continued tumor growth [46,47,48]. Fernand Labrie’s elegant work in this area for over two decades shed light on how, why, when, and the extent to which intracrine hormone synthesis occurs [46,47,49,50]. Studies have shown that estradiol concentrations were significantly higher intra-tumorally compared to serum and that the levels did not differ between pre- and post- menopausal women [51]. Also, estradiol concentration was >2 fold higher in breast carcinoma tissues than in surrounding normal tissues [52]. Recchione et al. determined the serum and tumor levels of estradiol, testosterone, and DHT in 34 patient specimens [53]. While the levels of testosterone were comparable between serum and tumor tissues, the concentration of estradiol and DHT was much higher in the tumor tissues than in blood [53]. In addition, cancers of the breast and prostate overcome pharmacological inhibition by synthesizing hormones through unconventional pathways [54,55,56,57,58]. These data support the importance of intracrine hormone synthesis in breast cancer.

**Figure 1:**
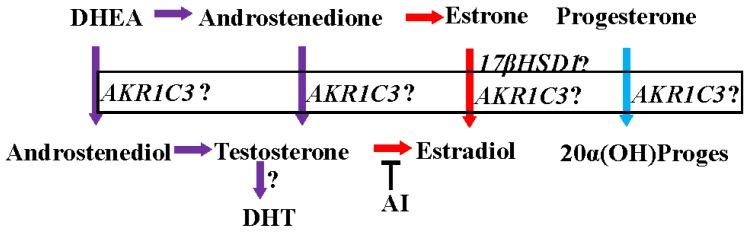
Intracrine synthesis of androgens, estrogens, and progesterone. AI: aromatase inhibitor; ?: functional importance in clinical breast cancer is not clear.

The activation and inactivation of steroid hormones are influenced by a class of enzymes called hydroxysteroid dehydrogenases (HSD), which catalyze the NAD(P)(H)-dependent oxidoreduction of the hydroxyl/keto groups of androgens, estrogens and their precursors [59,60] and thereby regulate the intracellular availability of steroid hormone ligands to their receptors [61]. HSDs modify the 3, 5, 11, 17, or 20 positions of the steroid backbone [61,62,63]. Fourteen of these enzymes are classified as mammalian 17β-HSDs [59]. Between 75% and 100% of circulating estradiol in pre- and post- menopausal women, respectively, is synthesized from adrenal precursors by steroidogenic enzymes (i.e., the 17-βHSD family and aromatase) [46,64]. One of the fourteen 17-βHSDs important for the activation of adrenal precursors is aldo keto reductase 1C3 (17-βHSD5 or AKR1C3). AKR1C3 converts estrone to estradiol, androstenedione (A′dione) to testosterone, and progesterone to 20α-hydroxy progesterone [65,66,67] (Figure 1).

Estrogens in pre-menopausal and post-menopausal women are synthesized from their adrenal androgen precursors, dihydroepiandrosterone sulphate (DHEAS) and dihydroepiandrosterone (DHEA) [46]. DHEAS and DHEA are converted to androstenedione (4′dione) and then to highly active androgens and estrogens in peripheral tissues. Tumor protective functions have been attributed to these adrenal androgen precursors. On one hand, low circulating levels of DHEA and DHEAS have been found in patients with breast cancer [68]. On the other hand, administration of DHEA and maintenance of serum DHEA levels similar to that of healthy pre-menopausal women resulted in significant inhibition of mammary carcinogenesis in rats [69]. Further, DHT was detected at higher concentrations in breast cancer tissues [53], supporting the hypothesis that a combination of AR expression and higher DHT levels are associated with a favorable prognosis in AR-expressing breast cancer tissues.

Together, these lines of evidence suggest that intracrine androgen synthesis, higher androgen concentrations, and AR expression are strongly associated with a better prognosis, favorable therapeutic outcome, and a reduction in tumor in patients with AR-positive breast cancers.

## 8. AR as Therapeutic Target for Breast Cancer

Steroidal androgens were the mainstay of clinical treatment for breast cancer before the discovery of tamoxifen or other ER antagonists and AIs [70,71]. Early preclinical evidence for the anti-proliferative effects was generated in 1950s when Huggins and colleagues showed shrinkage of chemically-induced mammary tumors by ovariectomy or by the administration of DHT, long before either the ER or AR had been cloned [72,73,74]. However, the use of androgens was discontinued after the discovery of ER antagonists or selective estrogen receptor modulators (SERMs) and AIs, owing largely to the undesirable masculinizing effects of steroidal androgens and the commercial promise of the newer therapies.

Despite or perhaps because of plentiful historical evidence, a controversy remains with respect to whether an AR agonist such as an androgen or an AR antagonist will be effective in treating breast cancer. The conflict is primarily due to the skewed outcome of experiments performed with preclinical immortalized cell line models. Below we summarize the clinical and preclinical evidence supporting the use of both AR agonists and antagonists as treatment options for breast cancer.

## 9. Preclinical Evidence Supporting the Beneficial Effects of AR agonists in Breast Cancer

Preclinical models to evaluate the role of the AR in breast cancer are highly variable. ZR-75-1 is an ER-positive luminal A breast cancer cell line that expresses high levels of the AR. Treatment of this cell line with DHT resulted in significant growth inhibition [75]. DHT inhibited both estradiol-dependent and estradiol–independent growth completely [75]. Unlike other cell lines, ZR-75-1 responds to physiologically relevant concentrations of DHT. These anti-proliferative effects were reversed by hydroxyflutamide, an AR antagonist. These in vitro results were extended in vivo in ovariectomized, estradiol-supplemented, nude mice bearing ZR-75-1 tumors [76]. In this study, DHT completely inhibited the tumor growth and even regressed the tumors. Due to very slow growth properties of ZR-75-1 cells, which is characteristic of ER-positive luminal A tumors, it is difficult to conduct xenograft studies in this model.

Tilley and colleagues using MCF-7 and T47D ER- and AR- positive luminal breast cancer cell lines demonstrated that two steroidal androgens (DHT and mibolerone) inhibited the cell proliferation [77]. Although the inhibition of proliferation was not as robust as that obtained in ZR-75-1 cells, the inhibition was also reversed by AR antagonists [77]. The differences in the magnitude of effects between cell lines could be due to the level of AR expression. MCF-7 cells have relatively lower AR expression compared to ZR-75-1 cells. Studies have also shown that androgens induce apoptosis in MCF-7 cells. On the other hand, some studies have also reported growth-stimulatory effects of androgens in modified MCF-7 cells [78]. Although these results define the variability in cell-based models, predominantly anti-proliferative effects were observed with androgens in ER- and AR-positive cells.

More convincing results evolved from the dimethylbenzanthracene (DMBA)-induced mammary carcinogenesis rat model [76]. Rats bearing DMBA-induced mammary tumors regressed significantly when treated with either strong androgens such as DHT or with weak androgen precursors such as DHEA, DHEAS, or 4′dione [69,76]. All these in vitro and in vivo results in multiple models unequivocally prove that AR agonists are inhibitors of ER-positive luminal breast cancers.

When analyzing the preclinical data in TNBC or BLBC models, the landscape is complex and inconclusive. Most of the data were generated in one ER-negative apocrine breast cancer cell line, MDA-MB-453. The proliferation of MDA-MB-453 cells or growth of MDA-MB-453 xenografts are stimulated by androgens and inhibited by AR antagonists [33,79]. It is yet unclear if the mutation in the AR LBD, p53, and PTEN, and constitutive activation of PIK3CA contribute to this phenotype of the cells [34,80]. However, ectopic expression of wildtype AR in MDA-MB-231 ER-negative cells restored the growth inhibitory effects of steroidal androgens and selective androgen receptor modulators (SARMs), which could be partially reversed by AR antagonists [79].

Barton et al. used TNBC cell lines to evaluate the effect of DHT [81]. Treatment of SUM159PT, HCC1806, BT549, and MDA-MB-231 cells with 10 nM DHT increased the proliferation of only SUM159PT, but not the other cell lines, while the proliferation of all cell lines was inhibited by enzalutamide, a nonsteroidal AR antagonist. The induction of proliferation by DHT in SUM159PT cells was modest. For unknown reasons, the proliferation of BT549 cells, which express AR at a level comparable to that of SUM159PT, was not induced by DHT. Growth of all cell lines was inhibited by AR antagonist enzalutamide or AR siRNA.

Ince and colleagues evaluated the effect of DHT in various ER-negative and TNBC cell lines [82,83]. While 10 nM DHT inhibited the proliferation of AR-positive CAL-148, MFM-223, and BT-474 in 8–10 days, DHT failed to inhibit the proliferation of AR-negative MDA-MB-468, SUM-159PT, or BT-20 cells. This group also evaluated the AR antagonist enzalutamide in these cell lines; some of which express the AR and some of which do not express the AR [82]. While enzalutamide inhibited the proliferation of prostate cancer cell lines with a 5-fold difference in IC_50_ values between AR-positive and -negative prostate cancer cell lines, it inhibited TNBC cell lines at comparable concentrations regardless of the AR expression. These results suggest that the effect of AR antagonist enzalutamide in TNBC cell lines could be AR independent.

Multiple lines of evidence suggest that the AR is a favorable prognostic indicator in breast cancer and that AR agonists would be the preferred approach for choice of androgenic treatment for ER-positive breast cancer. However, data is conflicting in TNBC. With multiple players involved in TNBC, the action of the AR in TNBC appears to be influenced by cross-talk with other pathways that differ between cell types and cancer subtypes.

## 10. Clinical Evidence Supporting the Use of AR Ligands in Hormone-Receptor-Positive Breast Cancer

Clinical evidence supporting the use of steroidal androgens for breast cancer dates back to the 1940s when testosterone and DHT were used to treat women with breast cancer [71,84]. Several studies using natural androgens demonstrated that the breast cancers regressed by 30%–50% in pre- and in post-menopausal women and that these effects were predominant in breast cancers expressing the AR [85,86,87,88]. Tumor growth regression with androgens was also observed after the removal of the pituitary, establishing that the effect of androgens is mediated directly through the AR expressed in the breast cancer tissue rather than through an effect on the hypothalamus pituitary hypogonadal axis [85].

Initial evidence of synthetic steroidal androgens showing growth inhibitory effects in breast cancer came from the use of fluoxymesterone (Halotestin™) and medroxyprogesterone acetate [89,90,91]. These synthetic androgens were not only effective in eliciting breast cancer regression, but were also effective in providing additive effects in combination with tamoxifen, providing a survival advantage to patients [92]. Although medroxyprogesterone has PR activity, it was effective in TNBCs that do not express PR, suggesting that the effects were achieved by through the ability of medroxyprogesterone to activate the AR [93].

Despite the historic and positive clinical results achieved with androgens in breast cancer, there have been few controlled clinical trials. As such, it remains unclear which subtypes respond best to androgens and the magnitude of response that can be expected. Ongoing clinical trials with newer nonsteroidal SARMs and nonsteroidal antiandrogens are poised to fill this knowledge gap. DHT, testosterone, and fluoxymesterone are steroidal androgens that have androgenic effects not only in breast, but also in other tissues including uterus, ovaries, skin, and hair follicles. SARMs were first reported in the late 1990s and subsequently shown to tissue selectively activate the AR in breast, muscle, and bone, without side effects associated with steroidal androgens [94,95,96,97,98]. Clinical trials with enobosarm (a nonsteroidal SARM being developed by GTx, Inc., Memphis, TN, USA) are ongoing to evaluate its efficacy and safety in breast cancer [94,95]. A phase II proof-of-concept clinical trial in 18 ER- and AR-positive breast cancer demonstrated a favorable response of stable disease in 42% of the evaluable patients. Since all the patients had bone-only disease, partial response or complete response could not be achieved. These results were presented at the San Antonio Breast Cancer Conference in 2015. Currently, enobosarm is being tested in Phase II clinical trials in subjects with ER-positive breast cancer and TNBC (NCT02463032 and NCT02368691, respectively). These early clinical results corroborate the clinical utility of androgens in breast cancer and suggest that nonsteroidal SARMs without the side effects commonly associated with steroidal androgens could provide a new avenue of hormonal therapy for certain subtypes of breast cancer.

Abiraterone acetate is an inhibitor of Cyp17A1 enzyme, an enzyme upstream in the steroidogenesis pathway. An intriguing result was obtained in a clinical trial with abiraterone in ER-positive breast cancer patients [99]. The central hypothesis for the study was that a complete inhibition of androgen and estrogen signaling would provide a better response in breast cancer. In this trial, 297 patients were stratified into three arms; with one arm receiving 1000 mg abiraterone plus 5 mg prednisone, one arm receiving 25 mg exemestane alone and one arm receiving exemestane and abiraterone [99]. The primary end-point was PFS. No significant difference in PFS was observed when abiraterone was combined with exemestane. The investigators found an increase in serum progesterone levels, which they believe could have contributed to the lack of clinical activity with abiraterone. However, recently a publication reported a protective effect of progesterone in breast cancer [100]. This has to be mechanistically further evaluated to understand why abiraterone did not provide a better outcome in both ER-positive breast cancer and in TNBC, while enzalutamide did in a TNBC clinical trial.

## 11. Clinical Evidences Supporting the Use of AR Ligands in ER-Negative Breast Cancer

The results obtained in MDA-MB-453 cells provided an impetus to evaluate antagonists in breast cancer, TNBC in particular. Two AR antagonists, bicalutamide and enzalutamide, and a CYP17A1 inhibitor, abiraterone, are currently used in the clinical treatment of prostate cancer. Repurposing these drugs to treat TNBC should prove straightforward if they are found to be effective in the clinic. An investigator-initiated clinical trial was conducted to evaluate the efficacy and safety of bicalutamide [101]. Out of the 424 patients with TNBC screened to determine the AR expression, only 51 were found to express AR. The trial treated 26 subjects with 150 mg bicalutamide daily. Although there were no partial or complete responses in the study, stable disease was observed in two patients for up to 6 months and five patients for greater than 6 months with a clinical benefit rate (CBR) of 19%. Although a modestly favorable response to bicalutamide was observed, it was interesting that subjects with tumor specimens that stained strongly for the AR were the least responsive to the drug while subjects with tumor specimens that stained very weakly for AR demonstrated the most durable responses.

A follow-up case report of one patient with AR-positive TNBC who relapsed after chemotherapy and progressed after multiple treatments and surgery and responded to treatment with 150 mg bicalutamide has also been published [102]. The patient achieved a complete response according to RECIST 1.1 criteria after 4 months of treatment and responded as long as 12 months when the report was published.

Based partially on the modest success achieved with bicalutamide, clinical trials in TNBC and ER-positive breast cancer were initiated with a second generation AR antagonist, enzalutamide. Enzalutamide has a unique mechanism of action where it blocks AR nuclear translocation and is more potent than bicalutamide [103]. Although no publications have come out on the trial, data presented in San Antonio breast cancer conference in 2014 and 2015 and in American Society for Clinical oncology (ASCO) 2015 annual meeting indicated a favorable response, including partial and complete responses, in approximately 40% of the patients. Details will emerge when the data are published.

Abiraterone, the CYP17A1 inhibitor, was tested in 34 AR-positive TNBC patients [104]. Patients were treated with 1000 mg abiraterone combined with 5 mg prednisone. At 6 months a CBR of 20% was achieved, which included one complete response and five subjects with stable disease of greater than 6 months. The overall response rate was 6.7% with median PFS of 2.8 months, which was far less than that observed with enzalutamide. Table 2 has a summary of clinical data.

## 12. Mechanisms of Action of the AR in Breast Cancer

**Figure 2:**
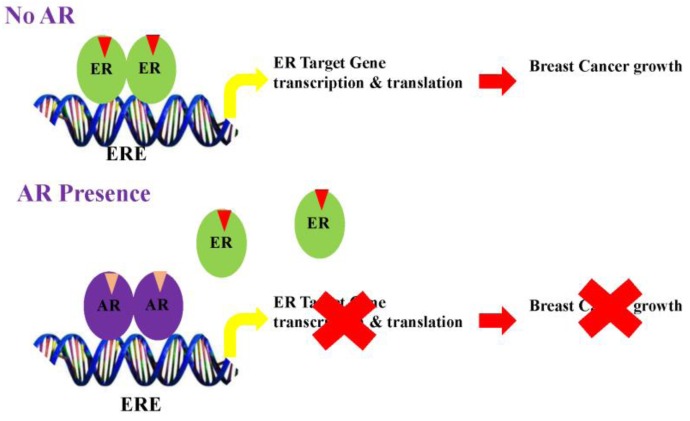
Mechanism for inhibition of estrogen receptor (ER)-positive breast cancer by the Androgen receptor (AR). (**A**) ER, in the presence of estrogens, binds to estrogen response elements (ERE) and activates the transcription and translation of target genes. AR, when activated by androgens, displaces ER and binds to EREs to form an inactive transcriptional complex, leading to inhibition of ER-target genes; (**B**) On the other hand, the AR, when activated by androgens, competes with ER for a limited pool of coactivators. This competition inhibits ER target genes and activates AR target genes. (Modified version of the figure published by McNamara et al. [25]).

Studies from several groups support the concept that AR elicits anti-proliferative effects in ER-positive breast cancers by antagonizing ER action. Data also suggests that the AR in the presence of agonists binds to estrogen response elements (EREs) by competing for common binding regions [105] (Figure 2). Likewise, gonist-activated AR may compete for the limited coactivator pool, thereby inhibiting ER function by sequestering coactivators from the ER.

While the mechanism is, safe to say, modestly clear in an ER-positive setting, it is still ambiguous in TNBC, especially considering that only one cell line MDA-MB-453 was used for mechanistic studies. The AR has been shown to cross-talk with several proteins in MDA-MB-453 cells. FOXA1 regulates AR and ER DNA binding and has significant overlapping binding regions in MDA-MB-453 [106]. Similarly, androgens were shown to increase extracellular signal-regulated kinase (ERK) and HER2 signaling in TNBC. Evaluation of PIK3CA kinase mutation in TNBC specimens showed that 40% of the AR-positive and 4% of the AR-negative specimens had mutations and concurrent amplifications [107,108]. Considering that the MDA-MB-453 cell line also contains a PIK3CA mutation, combination of the AR antagonist and PI3K/mTOR inhibitors provided additional effects [108]. Androgens in the presence of the AR have also been shown to abrogate the interaction between epithelial cells and mesenchymal stem cells to inhibit the paracrine metastatic factors [79].

## 13. Conclusions

The AR is a favorable prognostic marker and a promising therapeutic target in breast cancer. In ER-positive breast cancer, the landscape is clear suggesting that androgens and in particular nonsteroidal AR agonists may provide beneficial effects. On the other hand, data on TNBC is conflicting with historical data favoring the use of agonists, data from enzalutamide clinical trials supporting antagonists, and data from abiraterone clinical trials suggesting that inhibition of AR signaling is not beneficial. This is likely to come down to the subtypes in TNBC where a subtype might respond to agonists, while another subtype might respond to antagonists. A clear picture can be obtained only with new preclinical translational models such as the patient-derived xenografts (PDXs) that will provide clarity. Even in this case, the outcome and mechanisms might vary between patient specimens and exposure to prior treatments. In addition, the evolving AR splice variants (AR-SVs) have to be taken into consideration while planning a strategy [109]. Considering that splice variants lack the LBD, neither agonists nor antagonists that bind to the LBD are likely to provide a meaningful outcome. Similar to prostate cancer, prolonged treatment of patient’s specimen with enzalutamide resulted in an increase in the AR-SVs [109]. The AR-SVs in breast cancer is a nascent field requiring additional data before any direction could be chartered.

Overall the next few years, when results from clinical trials with enobosarm and enzalutamide will be available, are critical to provide greater clarity on the role of the AR in ER-positive and –negative breast cancers. Considering that new agonists and antagonists for the AR are available, the emergence of nonsteroidal drugs targeting the AR as a new hormonal treatment for breast cancer is almost certainly on the horizon.

## Figures and Tables

**Table 1:** Summary of studies showing the prognostic value of androgen receptor (AR) expression in breast cancer.

Reference	Ref	Summary
Pistelli et al., 2014	[29]	AR expression in TNBC (*n* = 81) was inversely correlated with Ki67 (*p* < 0.0001).
Vera-Badillo et al., 2014	[23]	A review of data from 19 studies that included 7693 women. AR expression was associated with improved OS and DFS (both in ER + ve and TNBC) at both 3 and 5 years *p* < 0.001).
Noh et al., 2014	[30]	334 ER − ve (HER2 + ve or −ve) cases were included in this study. AR − ve Her2 − ve patients were younger and had higher ki67 than AR + ve patients. Metabolic markers such as CAIX, which are associated with shorter DFS and OS, were lower in AR + ve Her2 − ve cancers
Sultana et al., 2014	[24]	Patients (in a study that included 200 women) with AR + ve tumors had higher OS. AR + ve ER-ve women had a trend for longer OS and encountered only 2 deaths (*n* = 16). On the other hand, AR − ve ER − ve women had shorter OS and had 10 deaths (*n* = 37).
McNamara et al., 2014	[25]	AR expression was associated with lower ki67, mostly TNBCs. AR was the only correlative marker for ki67 staining (lower)
McNamara et al., 2013	[37]	25% (51 samples) of 203 TNBC patients were AR + ve, 72% for 5-α reductase and 70% for 17βHSD5. AR negatively correlated with ki67. Co-expression of AR and androgenic enzymes negatively correlated with ki67 staining. AR − ve 5αR group had worse survival in an 80 month follow up.
Luo et al., 2010	[26]	Of 137 TNBC patients 38 were AR + ve. Of 132 non-TNBC patients 110 were AR + ve. AR + ve correlated with 5 year survival in TNBC, but not in non-TNBC.
Agoff et al., 2003	[28]	89% of ER + ve (*n* = 19) and 49% of ER − ve (*n* = 69) tumors were AR + ve. Patients with ER − ve and AR + ve tumors were older than AR − ve patients. AR − ve tumors had higher ki67 staining. ER − ve AR + ve tumors were lower grade, smaller and Her-2/neu over-expression. In ER + ve tumors AR positivity correlates with PR positivity. 84% of ER − ve, AR + ve patients were disease free after treatment, while only 53% of ER − ve, AR − ve patients were disease free after treatment. None of the ER-negative, AR-positive patients died, while 4 of ER-negative, AR-negative patients died.
Qu et al., 2013	[27]	109 breast cancer (ER + ve, ER − ve, TNBC) were included in this study. AR + ve breast cancers (all types) had better OS and DFS. AR was also associated with lower risk of recurrence.

**Table 2:** Summary of clinical data on AR agonists and antagonists in breast cancer.

Reference	Ref	Summary
Hermann and Adair, 1947, 1946	[71,84]	Treatment of patients with breast cancer with testosterone propionate showed significant regression of cancer and disappearance of metastases. Four out of 11 breast cancer patients treated with testosterone propionate exhibited favorable response.
Bines et al., 2014	[88]	Clinical trial with Megesterol acetate, a synthetic progestin that also has AR agonistic activity was conducted in ER-positive breast cancer patients. Clinical benefit rate of 40% was achieved with a duration of clinical benefit of 10 months.
Tormey et al.,1983	[90]	Combination of halotestin and tamoxifen was tested in a clinical trial conducted in ER-positive breast cancer patients. Combination was more effective with 38% partial and complete remission rates, while tamoxifen had only 15%. The duration of response was also longer in the combination group than in the tamoxifen group.
Gucalp et al., 2013	[101]	Clinical trial with an AR antagonist, bicalutamide, was performed in ER-negative breast cancer patients. The 6 month clinical benefit rate was 19% and the median PFS was 12 weeks. The drug was well tolerated.
Arce-Salinas et al., 2016	[102]	Case report of a patient with ER-negative breast cancer treated with bicalutamide. The patient showed a complete response and the response was also durable for over a year.
Bonnefoi et al., 2016	[104]	A clinical trial with abiraterone+prednisone in 30 AR-positive TNBC patients was performed. A clinical benefit rate of 20% was observed in this trial with an overall response rate of 6.7%.
O’Shaughnessy et al., 2016	[99]	Abiraterone acetate was tested alone or in combination with exemestane in patients with ER-positive breast cancer. There was no significant difference in the PFS in the combination arm compared to the exemestane arm.
